# SodA promotes immune evasion of *Streptococcus suis* by suppressing ROS accumulation and GSDMD-mediated mitochondrial disruption in neutrophils

**DOI:** 10.1128/spectrum.01901-25

**Published:** 2025-11-26

**Authors:** Honglin Xie, Yushu Li, Qiuguo Fang, Haoxian Xie, Jianyi Huang, Zhaoru Wu, Ziteng Deng, Qinqin Sun, Yunfei Huang, Jiedan Liao, Shun Li, Yajuan Li, Qiang Fu

**Affiliations:** 1 School of Animal Science and Technology, Foshan University 47868, Foshan, Guangdong, China; 2 Foshan University Veterinary Teaching Hospital, Foshan University 47868, Foshan, Guangdong, China; 3 State Key Laboratory for Sheep Genetic Improvement and Healthy Production, Institute of Animal Husbandry and Veterinary, Xinjiang Academy of Agricultural and Reclamation Science 418587, Shihezi, Xinjiang, China; Griffith University - Gold Coast Campus, Gold Coast, Queensland, Australia

**Keywords:** *Streptococcus suis*, neutrophil extracellular traps, mitochondria, GSDMD-N

## Abstract

*Streptococcus suis* is a major swine pathogen that poses a serious threat to pig health. Resistance to oxidative stress and modulation of host immune responses are both critical for the survival of *S. suis* serotype 2 (SS2) strains during infection. In this study, we investigated the role of the *sodA* gene in SS2 survival, neutrophil responses, and mitochondrial function, focusing particularly on neutrophil extracellular trap (NET) formation. Using a murine peritoneal infection model, we found that *sodA* deletion significantly reduced neutrophil recruitment. *In vitro* assays with primary mouse neutrophils further demonstrated that the *sodA* mutant exhibited reduced intracellular survival and elevated levels of reactive oxygen species (ROS) in neutrophils. The mutant also triggered more robust NET formation, as indicated by significantly increased levels of cell-free DNA and MPO–DNA complexes. Importantly, the inclusion of a complemented strain confirmed restoration of ROS clearance and normalization of NETs-associated markers, supporting the specificity of the *sodA*-dependent phenotype. Moreover, *sodA* deficiency exacerbated SS2-induced mitochondrial membrane depolarization and ROS accumulation in neutrophils. Notably, it also enhanced the expression of cleaved gasdermin D (GSDMD-N), which colocalized with mitochondria and likely contributed to mitochondrial damage and NET induction. Collectively, these findings suggest that *sodA* facilitates SS2 immune evasion by suppressing host ROS accumulation and GSDMD-N-mediated mitochondrial disruption, thereby limiting NET formation and promoting bacterial survival.

**IMPORTANCE:**

*Streptococcus suis* is a major swine pathogen with significant economic and zoonotic implications. Neutrophil extracellular traps (NETs) are essential for host defense, but their regulation by bacterial factors remains poorly understood. This study identifies the superoxide dismutase gene *sodA* as a key factor in immune evasion by *S. suis*. We demonstrate that *sodA* deletion enhances reactive oxygen species accumulation, mitochondrial damage, and NETs formation in neutrophils, impairing bacterial survival. These findings reveal a novel mechanism by which *S. suis* modulates host innate immunity and highlight *sodA* as a potential target for enhancing host defense against *S. suis* serotype 2 infection.

## INTRODUCTION

*Streptococcus suis* is an important zoonotic pathogen that causes invasive infections in pigs and has emerged as a public health concern due to its potential to infect humans and other mammals (1). In humans, *S. suis* infection can result in severe clinical outcomes such as septic shock, meningitis, and sudden death (2, 3). Notably, two major outbreaks of human *S. suis* serotype 2 (SS2) infections occurred in China in 1998 and 2005, resulting in 229 confirmed cases and 52 fatalities (4). *S. suis* can evade host innate immune defenses, allowing it to disseminate systemically (5). Numerous virulence factors have been identified that facilitate bacterial survival and host colonization. However, the molecular mechanisms underlying *S. suis* pathogenicity and immune evasion remain incompletely understood (6). Further investigations are therefore needed to elucidate the specific roles of these virulence factors in modulating host immune responses during infection.

Superoxide dismutase (SOD) is a key antioxidant enzyme that protects cells from reactive oxygen species (ROS), thereby affecting the occurrence and development of diseases (7). In bacteria, SODs are metalloenzymes that can be categorized into three main isoforms based on their metal ion cofactors: MnSOD, FeSOD, and CuZnSOD, encoded by the *sodA*, *sodB*, and *sodC* genes, respectively (8). In SS2, SOD activity is conferred by the *sodA*-encoded MnSOD (9), which protects bacteria from oxidative killing during infection. In contrast, the host also utilizes SODs as part of its antimicrobial defense. The mitochondrial isoform SOD2 can be trafficked via mitochondria-derived vesicles to bacteria-containing phagosomes in macrophages, where it promotes hydrogen peroxide production and facilitates bacterial clearance (10). Interestingly, recent evidence shows that bacterial MnSODs may exert broader regulatory functions. The MnSOD encoded by *sodA* in *Stenotrophomonas maltophilia* has been identified as a c-di-GMP effector protein, linking second messenger signaling to oxidative stress tolerance (11).

Neutrophils are among the first immune cells to respond to infection and play a pivotal role in host defense against pathogenic microorganisms (12). One of their key antimicrobial strategies is the formation of NETs, which are web-like structures composed of decondensed chromatin coated with histones and proteolytic enzymes. These structures trap and directly kill invading pathogens, representing an important component of the innate immune system (13, 14). However, SS2 has evolved multiple mechanisms to evade or resist NET-mediated killing, thereby promoting its survival and dissemination within the host (15, 16).

Gasdermin D (GSDMD) belongs to the gasdermin family and plays a critical role in the formation of cytoplasmic membrane pores. It can be cleaved by caspase 1/11 to form the GSDMD N-terminal domain (GSDMD-N) (17). Beyond its canonical role in pyroptosis, GSDMD has recently been implicated in neutrophil extracellular trap (NET) formation. Cytosolic lipopolysaccharide and intracellular gram-negative bacteria can activate the caspase-11/GSDMD pathway in neutrophils, leading to GSDMD-dependent NET release and protection against cytosolic infection (18). Activated GSDMD also induces rapid, cardiolipin-dependent mitochondrial damage, resulting in increased production of ROS (19). Mitochondrial respiration has been identified as a key contributor to NETs formation, and mitochondrial ROS (mtROS) are essential for spontaneous NET release in low-density granulocytes (20, 21).

Recent studies further indicate that GSDMD-N can form pores in mitochondrial membranes, leading to the leakage of mitochondrial DNA (mtDNA) and promoting a feed-forward loop of mtROS production and NETs extrusion (19). Moreover, neutrophil proteases such as elastase and cathepsin G can also cleave and activate GSDMD, amplifying chromatin decondensation and NET release (22).

In the case of SS2, the recognition by TLR2 and/or TLR4 initiates NETs formation via NADPH oxidase–derived ROS signaling. These ROS activate downstream MAPK pathways, such as p38 MAPK and ERK1/2, ultimately driving NETs release (23). Meanwhile, the *sodA* gene of SS2 has been shown to suppress ROS production in phagocytes by enhancing autophagic activity (24), suggesting its potential role in modulating NETs formation. These observations suggest a potential mechanistic link between bacterial *sodA*-mediated redox regulation and host GSDMD/mitochondrial-driven NETs formation, which remains largely unexplored and forms the basis of the present study.

In this study, we investigated the role of the *sodA* gene in SS2-induced NETs formation. Using a *sodA* deletion mutant, we found that *sodA* deficiency enhanced ROS production, mitochondrial damage, and NETs release in neutrophils. Furthermore, the complemented strain confirmed that *sodA* contributes to ROS clearance and suppresses excessive NETs formation. Moreover, increased expression and mitochondrial localization of GSDMD-N suggest its involvement in NETs induction. These findings provide new insights into the mechanism by which *sodA* contributes to SS2 immune evasion and adaptation to the host environment.

## MATERIALS AND METHODS

### Bacterial strains and experimental animals

The wild-type (WT) SS2 strain ZJ081101 (serotype 2) and *sodA* deletion mutant (Δ*sodA*) are obtained from Zhejiang University laboratory (25). The WT and Δ*sodA* strains were cultured in a Tryptose Soya Broth (TSB, Oxoid, UK) containing 5% newborn calf serum or plated on Tryptose Soya Agar (TSA; Oxoid, UK) containing 5% newborn calf serum.

The complemented strain (C*ΔsodA*) was constructed based on previously described methods (25). Briefly, the *sodA* gene, including its native promoter region, was amplified and cloned into the shuttle vector pSET2, followed by electroporation into the Δ*sodA* mutant. Recombinant colonies were confirmed by sequencing, and *sodA* transcription was validated by RT-PCR and qPCR (Fig. S1). The primers used in this study are listed in Table S1.

C57BL/6 mice were used for all *in vivo* experiments. Mice were housed in specific pathogen-free (SPF) facilities at South China Agricultural University (Guangzhou, China). All experiments were conducted using 6-week-old, sex-matched mice. Animal studies were approved by the Animal Welfare and Research Ethics Committee of Guangdong Province (approval number: SYXK [Guangdong] 2019-0136).

### Neutrophil isolation and identification

Neutrophils were isolated from the bone marrow of 6-week-old SPF mice. Briefly, bone marrow cells were flushed from the tibias and femurs using sterile RPMI 1640 medium and a 1 mL syringe and collected into 15 mL centrifuge tubes. The cell suspension was layered over Ficoll-Hypaque (Solarbio, P8550) and centrifuged at 900 × *g* for 30 min at room temperature. The upper layer containing lymphocytes and monocytes was removed, and the granulocyte-rich layer was collected while avoiding the erythrocyte pellet at the bottom. The collected cells were washed with RPMI 1640 medium. All procedures were performed under sterile conditions at room temperature. Cell viability was assessed using Trypan Blue staining, which showed that neutrophils remained viable for up to 5 h after isolation. The purity of isolated neutrophils (>90%) was confirmed by flow cytometry (Fig. S2).

### Intraperitoneal infection and peritoneal lavage fluid collection

SS2 strains were cultured in TSB medium supplemented with 5% newborn calf serum at 37°C until they reached the mid-logarithmic phase. Viable SS2 counts were obtained by plating serially diluted samples onto TSA. For infection experiments, mice were intraperitoneally inoculated with WT and Δ*sodA* at a dose of 1 × 10⁹ CFU in 500 µL of PBS, and an equivalent volume of sterile PBS was similarly administered intraperitoneally to the control group (ctrl). After injection for 12 h, the mice were sacrificed, and 1 mL of PBS was injected into the peritoneal cavity. Gentle massage of the abdomen for 5 min and drawing back the peritoneal lavage fluid (PLF). PLF was detected using an Auto Hematology Analyzer (BC-5000 Vet; Mindray, China), and flow cytometry was used to determine the proportion of neutrophils.

### Phagocytosis rate and intracellular survival rate

Neutrophils were co-incubated with SS2 at a multiplicity of infection (MOI) of 10 for 2 h at 37°C. Following incubation, the supernatant was removed, and 1 mL of RPMI 1640 medium containing 20 µg/mL gentamicin was added to each well to eliminate extracellular bacteria. After 1 h of incubation, the medium was discarded, and neutrophils were washed three times with ice-cold PBS. To quantify phagocytosed bacteria, neutrophils were lysed with ultrapure water, and the lysates were serially diluted and plated on TSA agar. CFUs were counted after overnight incubation. The phagocytosis rate was calculated as: (CFU in neutrophil lysate/CFU in original inoculum) × 100%.

For intracellular survival analysis, neutrophils were incubated for an additional 1 h after gentamicin treatment. Cells were then lysed and plated as described above. The intracellular survival rate was calculated as: (CFU after 1 h culture/CFU immediately post-gentamicin treatment) × 100%. The detailed protocol was adapted from previously published methods (26).

### Neutrophils and NETs bactericidal assays

As previously described (27), the neutrophils were divided into two groups: an untreated group containing only neutrophils, and a group of neutrophils treated with 100 U/mL DNase I (Sigma) to inhibit the formation of NETs. WT or Δ*sodA* at 2 × 10^7^ CFU were added to neutrophils (MOI = 10). After incubation with WT or Δ*sodA* for 2 h, the cell supernatant was collected, and neutrophils were permeabilized with ultrapure water on ice to release the intracellular bacteria. The supernatant and neutrophil lysate were mixed and plated on TSA agar plates, and CFUs were counted. The initial inoculum was quantified by serially diluting and plating bacteria that had not been incubated with neutrophils. The percentage of surviving bacteria was subsequently calculated as (CFU supernatant and neutrophil lysate/CFU original inoculum) × 100%.

Neutrophils were stimulated with phorbol 12-myristate 13-acetate (PMA, 400 nM, Sigma-Aldrich) for 4 h to form NETs, as previously described (28). The mixtures were then centrifuged at 500 × *g* for 5 min to isolate the cell supernatant. The supernatant containing NETs is henceforth referred to as NETs. WT and Δ*sodA* strains at 3 × 10^6^ CFU were incubated with NETs for 1.5 h at 37°C. Surviving bacteria were counted by plating the serially diluted samples on TSA agar plates. The SS2 survival rate in NETs was calculated as (CFU survival bacteria/CFU original inoculum) × 100%.

### Assessment of the proportion of neutrophils of PLF and mitochondrial membrane potential by flow cytometry

The proportion of neutrophils in PLF was determined by specific staining of neutrophils. Neutrophils were stained with 0.1 µg of PE-mouse Ly6G antibody (eBioscience, 12-5931-81) and 0.1 µg of FITC-mouse CD11b antibody (eBioscience, 11-0112-82).

A total of 4 × 10^6^ neutrophils (2 × 10^6^ cells/mL) were seeded per well in a six-well plate, stimulated with RPMI 1640 medium, WT, Δ*sodA*, or 400 nM PMA for 2 h at 37°C and 5% CO_2_. Neutrophils in RPMI 1640 medium (without phenol red) stained with 200 nM MitoTracker Red (Biyotime) and 50 nM 2,7-Dichlorodihydrofluorescein diacetate (DCFH-DA, Biyotime) for 30 min at 37°C, followed by washing in PBS. Flow cytometry was used to analyze the cell suspensions. All experiments were performed using the CytExpert software and analyzed using the FlowJo software.

### Quantification of NETs production

Cell-free DNA (cfDNA) was used to quantify NETs (29). Neutrophils were incubated for 2 h in RPMI 1640 medium, WT, or Δ*sodA* infection (MOI = 10). Neutrophils were treated separately with 400 nM PMA (Sigma-Aldrich) as a positive control. cfDNA was quantified by using the PicoGreen DNA kit (Invitrogen). Fluorescence was measured using a TECAN Multilabel Reader, with excitation at 480 nm and emission at 520 nm.

### Measurement of MPO-DNA in cell supernatant

Circulating NETs in cell supernatants were measured using ELISA to detect myeloperoxidase (MPO)-DNA complexes (30). Neutrophils were co-incubated with RPMI 1640 medium, WT, or Δ*sodA* for 2 h, and the cell supernatants were collected. MPO-DNA levels in cell supernatants were analyzed using an ELISA kit (MEIMIAN, China), according to the manufacturer’s instructions. The kit assay measures Mouse MPO-DNA levels in the sample by using a purified Mouse MPO-DNA antibody to coat the wells of a microtiter plate, thereby creating a solid-phase antibody. Subsequently, MPO-DNA was added to the wells, where it bound with HRP-labeled MPO-DNA antibodies to form an antibody-antigen-enzyme-antibody complex. After thorough washing, the TMB substrate solution was introduced; this substrate underwent a color change to blue as catalyzed by the HRP enzyme. The reaction was terminated by the addition of sulfuric acid, and the resulting color change was quantified at a wavelength of 450 nm. Finally, the concentration of mouse MPO-DNA in the samples was determined by comparing their optical density values against a standard curve.

### Immunostaining and confocal microscopy

Neutrophils (2 × 10^5^ cells/well) were seeded on coverslips in 24-well plates and incubated for 1 h. Neutrophils were treated with SS2 or PMA. The cells were then fixed with 4% paraformaldehyde for 30 min. The cells were then permeabilized with 0.1% Triton X-100 for 10 min at room temperature and blocked with 3% normal goat serum for 2 h. Cells were then incubated with the indicated primary antibodies (anti-MPO antibody, 1:200, Abcam, EPR20257; anti-neutrophil elastase [NE] antibody, 1:200, BIOSS, bs-6982R; anti-GSDMD-N antibody, 1:200, Abcam, EPR20829-408) overnight at 4°C, followed by incubation with the corresponding fluorescent-conjugated secondary antibody goat-anti-rabbit conjugated to Alexa Fluor 488 (1:150, yeasen, 33106ES60) for 2 h. Nuclei were stained with DAPI (Solarbio) for 10 min. Fluorescence staining of the mitochondrial membrane potential and intracellular ROS was performed using MitoTracker Red (Biyotime) and DCFH-DA. The coverslips were then washed and mounted using antifade mounting medium (Biosharp, BL701A) on microscopy slides (SETech). Images were captured using a Zeiss 880 Laser Scanning Confocal Microscope with a 20× or 63× oil immersion objective and analyzed using the Zeiss Zen software. Pearson’s correlation coefficient was analyzed using the Zeiss Zen software.

### Measurement of reactive oxygen species

The level of ROS in the neutrophils was detected using the DCFH-DA probe. Neutrophils coupled with DCFH-DA working solution were added to a 96-well plate and incubated at 37°C for 20 min, and the fluorescence density was measured using a TECAN Multilabel Reader at an excitation wavelength of 560 nm and an emission wavelength of 610 nm. ROS levels were normalized to the control group and expressed as fold change relative to untreated neutrophils.

### Western blotting

The method follows our previous research (31). Bone marrow–derived neutrophils were incubated with RPMI 1640 medium, WT, or ΔsodA strains for the indicated time periods. Cells were then lysed in RIPA buffer (Beyotime Biotechnology, China) to extract total protein. Protein concentrations were determined using a BCA Protein Assay Kit (Beyotime Biotechnology, China). Equal amounts of protein were separated by SDS-PAGE and transferred onto polyvinylidene difluoride membranes. Membranes were blocked with 5% bovine serum albumin for 2 h at room temperature and then incubated with the following primary antibodies diluted in universal antibody diluent (WB500D, Biotech): anti-GSDMD/GSDMD-N (1:1,000, Abcam, ab219800) and anti-β-actin (1:1,000, Abcam, ab8227). After three washes with TBST, membranes were incubated with HRP-conjugated goat anti-rabbit IgG secondary antibody (1:10,000, Abcam, ab205718) for 1 h at room temperature. Protein bands were visualized using enhanced chemiluminescence reagents (Solarbio, China) and detected using a gel imaging system (Tanon 4200, China). Densitometric analysis of protein bands was performed using ImageJ software.

### Statistical analysis

All data are presented as the mean ± SD. One-way ANOVA was used for data analysis, as detailed in the figure legends. Analyses were conducted using GraphPad Prism version 8.0.2 (GraphPad Software, La Jolla, CA). *P < 0.05; **P < 0.01; ***P < 0.001; ns, no difference between groups.

## RESULTS

### Deletion of *sodA* reduces neutrophil recruitment in the PLF of mice

**Fig 1:**
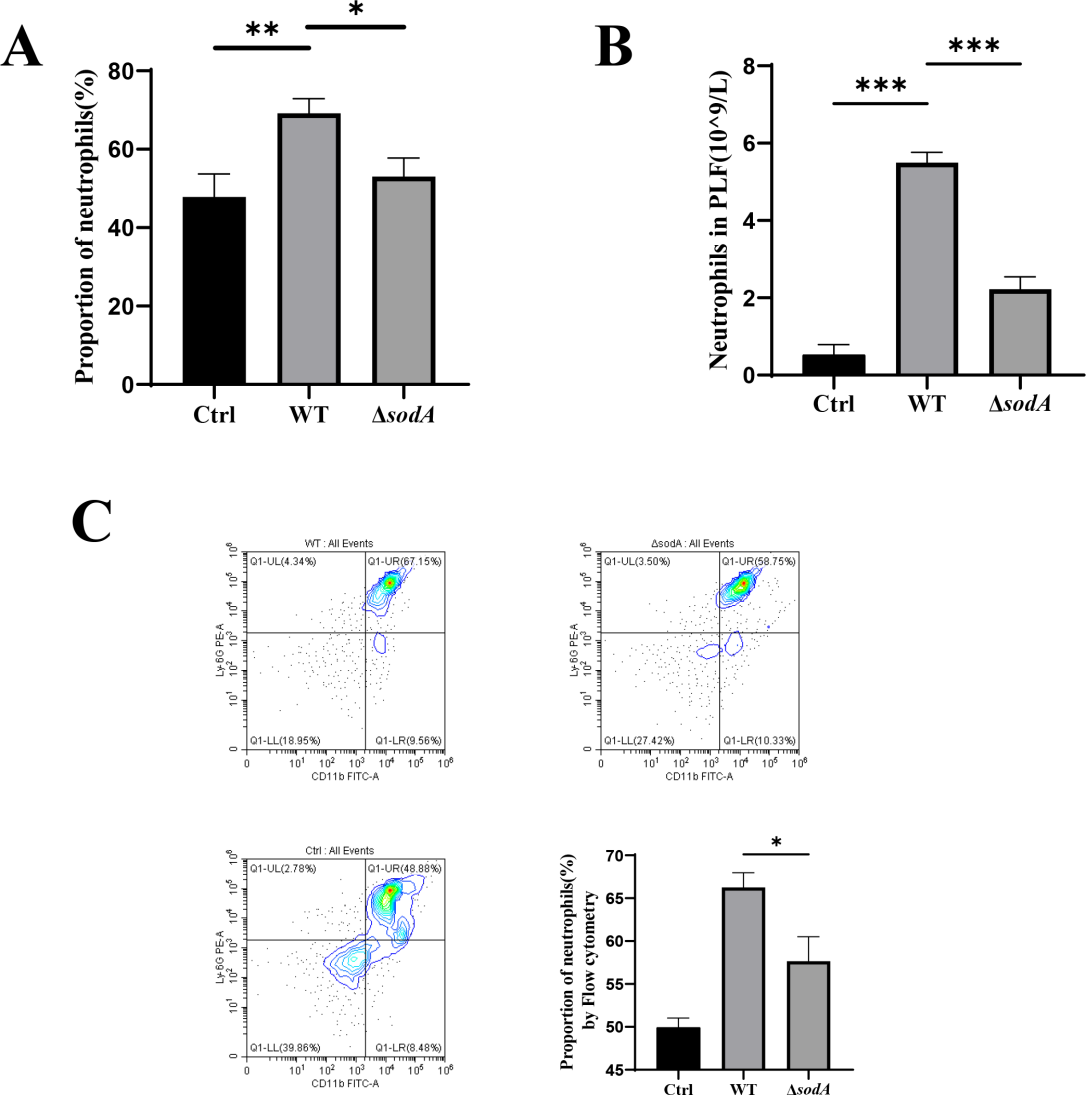
Neutrophil counts and proportions in PLF following WT and Δ*sodA* infection. (**A and B**) Total neutrophil counts (**A**) and proportions (**B**) in peritoneal lavage fluid, as determined using an automated hematology analyzer. (**C**) Neutrophils were identified as CD11b^+^/Ly-6G^+^ cells and analyzed by flow cytometry. Each group includes three mice (*n* = 3). *P < 0.05; **P < 0.01; ***P < 0.001.

We first established a murine peritoneal infection model by intraperitoneally injecting mice with either the WT or the Δ*sodA* strain. To assess neutrophil recruitment during the early phase of infection, PLF was collected 12 hours post-injection. Mice injected with PBS served as the control group. Neutrophil counts and proportions in PLF were measured using an automated hematology analyzer. Compared to the WT group, Δ*sodA* infection resulted in a significant reduction in neutrophil recruitment (Fig. 1A and B). Further confirmation was obtained by flow cytometry, where neutrophils were identified using CD11b and Ly-6G markers. The proportion of neutrophils was significantly lower in the Δ*sodA*-infected group compared to the WT group (Fig. 1C).

### *sodA* deficiency reduces the intracellular survival of SS2 in neutrophils

**Fig 2:**
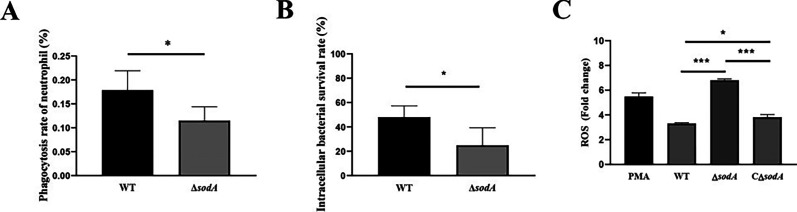
Determination of the phagocytosis and intracellular killing of SS2 by neutrophils. (**A**) Phagocytosis rates of WT and Δ*sodA* in the neutrophils. (**B**) Intracellular survival rates of WT and Δ*sodA* cells in the neutrophils. (**C**) Measurement of ROS fluorescence intensity after infection with WT, Δ*sodA*, and CΔ*sodA* strains. The results are presented as the mean ± SD (*n* = 4). *P < 0.05; ***P < 0.001.

We first evaluated the phagocytic uptake and intracellular survival of SS2 in neutrophils. Both the phagocytosis rate and the intracellular survival of the Δ*sodA* strain were markedly lower than those of the WT strain (Fig. 2A and B), suggesting that *sodA* is required for effective resistance to neutrophil-mediated killing.

ROS possess direct antimicrobial activity against intracellular bacteria (32). To explore the effect of *sodA* on host oxidative responses, we assessed ROS production in neutrophils following infection with WT, Δ*sodA*, and CΔ*sodA* strains. Compared with WT, Δ*sodA* infection induced significantly higher ROS levels in neutrophils, whereas ROS levels in the complemented strain were reduced relative to Δ*sodA* but remained slightly elevated compared with WT (Fig. 2A). These findings confirm that *sodA* contributes to the suppression of neutrophil ROS responses during SS2 infection.

### *sodA* deficiency enhances SS2-induced NETs formation

**Fig 3:**
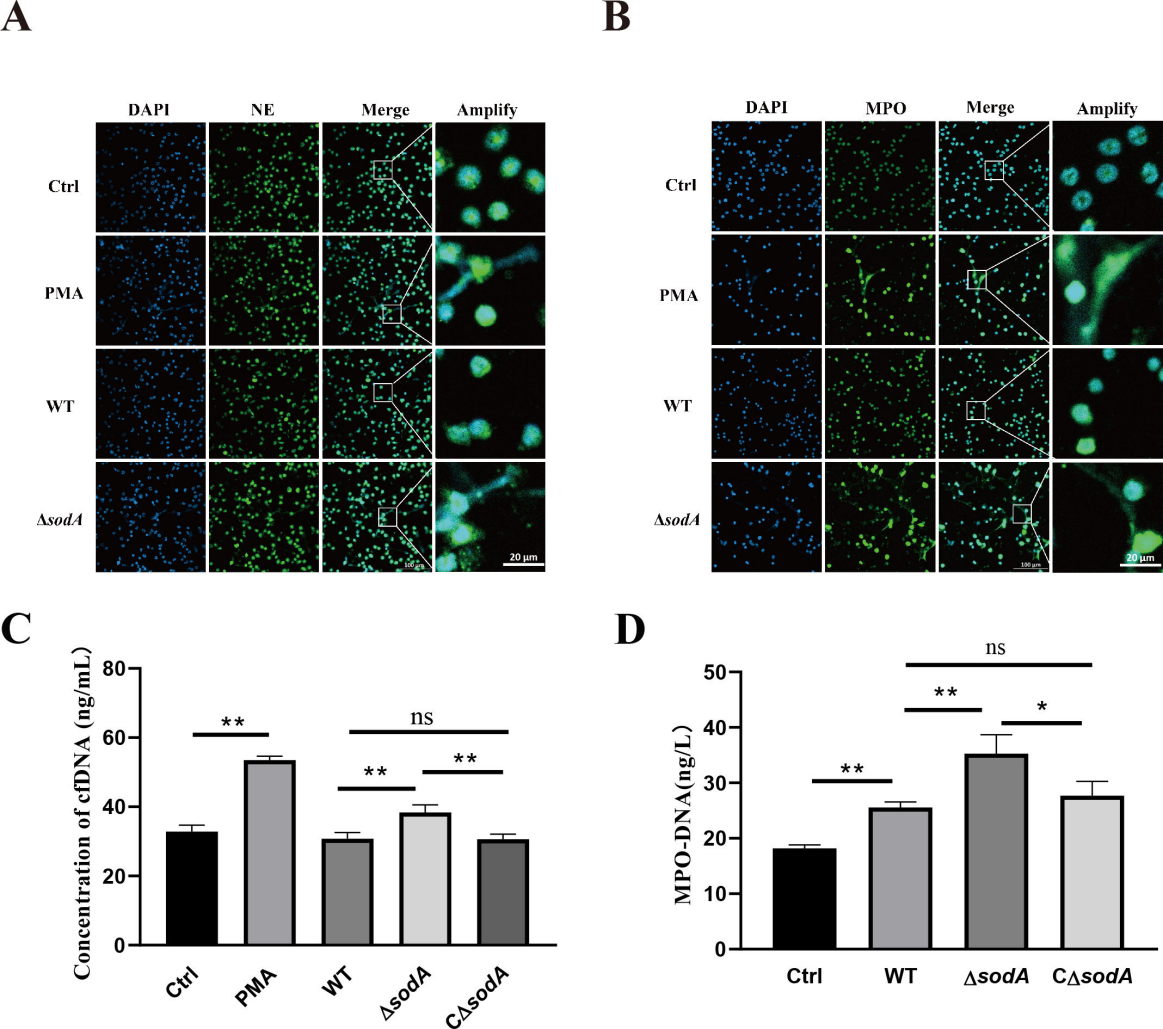
Visualization and quantification of NETs induced by SS2. (**A and B**) Neutrophils were incubated with WT, Δ*sodA*, or PMA (positive control) for 2 h. Immunofluorescence staining was performed using anti-NE or anti-MPO antibodies, followed by goat anti-rabbit IgG (H + L) conjugated with a green fluorophore. DNA was counterstained with DAPI (blue). (**C**) Quantification of cfDNA in the supernatants of neutrophils after 2 h of incubation with WT, Δ*sodA*, CΔ*sodA*, or PMA. (**D**) Quantification of MPO-DNA in the supernatants. Data are presented as mean ± SD (*n* = 3). *P < 0.05; **P < 0.01; ns, not significant.

To investigate whether *sodA* has an effect on SS2-induced NETs formation, WT and Δ*sodA* were infected with neutrophils, marked by neutrophil elastase or MPO antibody. Fluorescence microscopy revealed that neutrophils infected with Δ*sodA* for 2 hours exhibited extensive NET structures, whereas those infected with the WT strain showed minimal NETs formation (Fig. 3A and B).

We next quantified NETs markers among WT, Δ*sodA*, and CΔ*sodA* infections. Both cfDNA and MPO–DNA complex levels were significantly increased in neutrophil supernatants following Δ*sodA* infection compared with WT, whereas the complemented strain restored these levels close to those of WT (Fig. 3C and D). These results indicate that *sodA* deletion enhances SS2-induced NETs formation, and complementation of *sodA* reverses this effect, confirming that *sodA* negatively regulates NETs induction.

### *sodA* deficiency reduces SS2 resistance to NET-mediated killing

**Fig 4:**
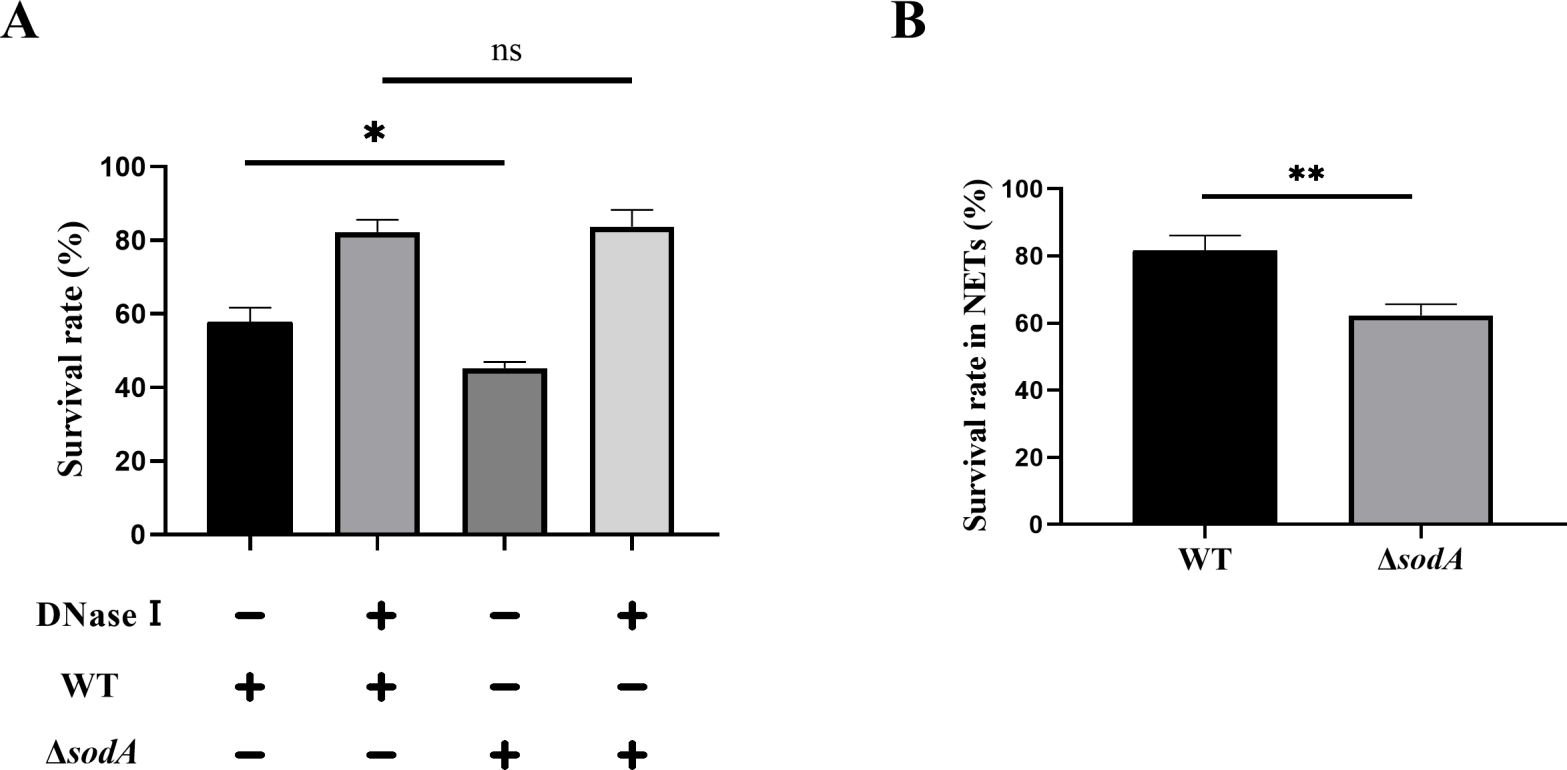
Bactericidal activity of neutrophils and NETs against WT and Δ*sodA* strains. (**A**) Survival of WT and Δ*sodA S. suis* strains after 2 h of co-incubation with neutrophils, with or without DNase I treatment. (**B**) Bactericidal effect of NETs on WT and Δ*sodA*. NET-rich supernatants were generated by PMA-stimulated neutrophils and incubated with bacteria at 37°C for 1.5 h. Viable bacterial counts were determined by plating. Data are presented as mean ± SD (*n* = 3). *P < 0.05; **P < 0.01; ns, not significant.

As NETs are known to play a key role in the neutrophil response to *S. suis* infection, we investigated whether *sodA* affects bacterial resistance to NET-mediated killing. Neutrophils were infected with either WT or Δ*sodA* strains for 2 h, and bacterial survival was assessed by plating the lysates on TSA plates. Compared to WT, the survival of the Δ*sodA* strain was significantly reduced (Fig. 4A). However, when DNase I was added to degrade NETs during incubation, this difference was abolished, indicating that the killing effect was NETs dependent. To further confirm this, neutrophils were stimulated with PMA to generate NET-rich supernatants, which were then incubated with WT or Δ*sodA* bacteria. The results showed that NETs exerted bactericidal activity against both strains, but Δ*sodA* was significantly more susceptible to NET-mediated killing than WT (Fig. 4B). These findings suggest that the deletion of *sodA* compromises the ability of *S. suis* to resist NET-mediated clearance.

### *SODA* deficiency increases mitochondrial membrane damage and ROS production in neutrophils infected with SS2

**Fig 5:**
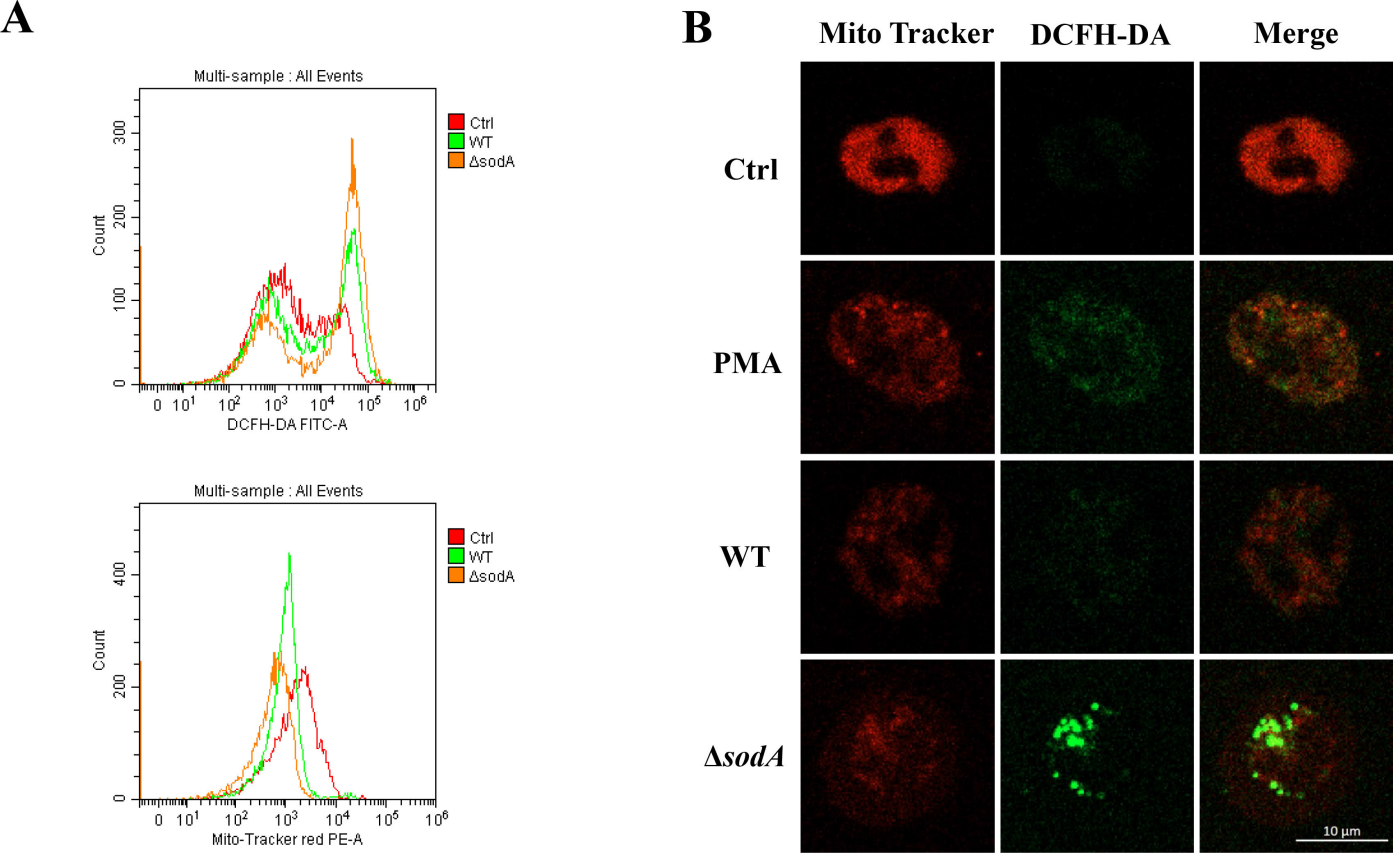
Δ*sodA* infection increases mitochondrial damage and ROS production in neutrophils. (**A**) Flow cytometry analysis of neutrophils stained with the ROS-sensitive dye DCFH-DA and MitoTracker Red after infection with WT or Δ*sodA* strains of SS2. (**B**) Live-cell imaging of neutrophils co-stained with DCFH-DA and MitoTracker Red showing changes in cellular ROS levels and mitochondrial membrane potential following infection.

Mitochondria are a major source of intracellular reactive ROS (33). To assess mitochondrial function and oxidative stress in neutrophils during SS2 infection, we measured cellular ROS levels and mitochondrial membrane potential. Compared to the WT strain, Δ*sodA* infection significantly increased ROS accumulation and reduced mitochondrial membrane potential in neutrophils (Fig. 5A and B). These results indicate that *sodA* deficiency exacerbates SS2-induced mitochondrial membrane damage and ROS production in neutrophils.

### *sodA* deficiency enhances GSDMD-N expression and its mitochondrial localization in neutrophils

**Fig 6:**
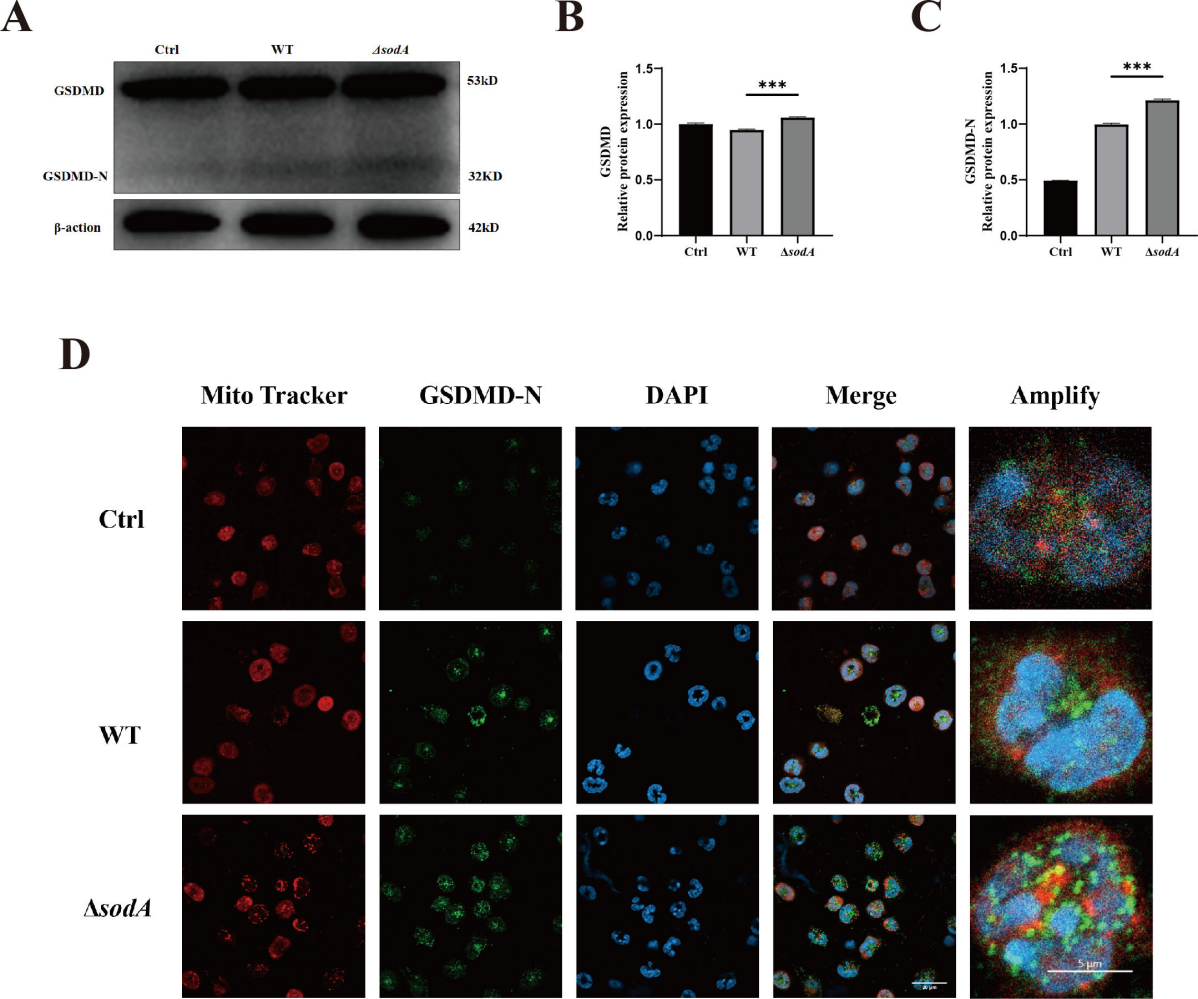
*sodA* deficiency enhances GSDMD-N expression and mitochondrial colocalization in neutrophils. (**A**) Western blot analysis of GSDMD and its cleaved form GSDMD-N in neutrophils after 2 h of infection with WT or Δ*sodA* strains. (**B and C**) Quantification of band intensities, normalized to β-actin, showing relative expression levels of GSDMD and GSDMD-N. (**D**) Immunofluorescence staining of neutrophils using anti-GSDMD-N antibody (green) and MitoTracker Red to assess mitochondrial colocalization. All experiments were performed in triplicate (*n* = 3). ***P < 0.001.

Activated GSDMD forms membrane pores that disrupt organelle integrity, including the mitochondrial membrane. To assess GSDMD activation during SS2 infection, we examined the expression of full-length GSDMD and its cleaved N-terminal fragment (GSDMD-N) in neutrophils by western blot. Both WT and Δ*sodA* strains induced GSDMD-N expression, but levels were significantly higher in the Δ*sodA* group (Fig. 6A through C). We further investigated the role of GSDMD-N in SS2 infection in neutrophils. In fluorescence microscopy images, GSDMD-N colocalized with and formed puncta on the mitochondria (Fig. 6D). We next investigated the subcellular localization of GSDMD-N during infection. Immunofluorescence microscopy revealed that GSDMD-N colocalized with mitochondria and formed distinct punctate structures on the mitochondrial surface (Fig. 6D). These results indicate that GSDMD is recruited to mitochondria during SS2 infection, and *sodA* deficiency enhances GSDMD-N production in neutrophils.

## DISCUSSION

*S. suis* is regarded as a leading infectious agent in the swine industry and also threatens human health (1). Generally, SS2 is considered to be the most virulent *S. suis* and is frequently isolated from clinically diseased piglets (34). To date, more than 100 “putative virulence factors or characteristics” of SS2 have been identified, at least 37 of which are considered critical for virulence (35). Neutrophils are key innate immune cells that play vital roles in host defense, and one of their major antimicrobial mechanisms is the formation of NETs (36). SS2 can secrete a variety of enzymes, such as SsnA, endA, and aptamers to inhibit the formation of NETs (37, 38). NETs formation is related to the production of ROS, both cytosolic and mitochondrial (21). However, the mechanism by which SS2 affects ROS production and induces NET formation requires further investigation.

Neutrophil recruitment is a critical component of the host immune response to *S. suis* infection. A previous study demonstrated that SS2 infection induces neutrophil accumulation at the infection site (39). Additionally, mice deficient in Fpr2 or NLRP6 exhibit enhanced early neutrophil recruitment and improved survival following SS2 infection (40), whereas vimentin deficiency suppresses neutrophil recruitment to the airway epithelium (41). In other streptococcal species, immune evasion mechanisms further illustrate the importance of neutrophil recruitment. For example, *Streptococcus pyogenes* secretes a platelet-activating factor esterase that impairs neutrophil recruitment and facilitates innate immune evasion (42). In SS2, the inactivation of the *sodA* gene reduces bacterial survival within macrophages and significantly attenuates virulence in mice (25), suggesting that *sodA* contributes to resistance against oxidative killing and may influence neutrophil-related immune responses.

Neutrophils exert direct bactericidal activity primarily through the production of ROS (43). However, many bacterial pathogens have evolved strategies to counteract ROS generation within neutrophils (44). One key mechanism involves the expression of SOD, which detoxifies superoxide radicals and helps bacteria survive oxidative stress (45). In the present study, neutrophils infected with the Δ*sodA* strain produced significantly higher levels of ROS than those infected with the WT strain. Moreover, Δ*sodA* was more susceptible to ROS-mediated killing (Fig. 2A through C). Importantly, the complemented strain (CΔ*sodA*) restored both ROS levels and bacterial survival to near wild-type levels, confirming that *sodA*-mediated oxidative stress resistance contributes directly to SS2 survival within neutrophils.

In addition to their direct bactericidal effects, ROS also contribute to the formation of NETs, representing another key mechanism by which neutrophils eliminate invading pathogens (17). ROS are typically generated downstream of pattern recognition receptor activation by pathogen-associated molecular patterns or damage-associated molecular patterns (DAMPs). They amplify innate immune signaling, particularly via NF-κB and inflammasome activation. Moreover, excessive ROS can promote the release of DAMPs such as mtDNA, establishing a feed-forward loop that sustains inflammation.

Several pathogens have evolved strategies to manipulate host ROS production and, in turn, NETs formation. For instance*, Clostridium perfringens* could influence the production of ROS by influencing arachidonic acid metabolism through the virulence gene phospholipase C and further influence the production of NETs (46). Likewise, *Burkholderia pseudomallei* regulates NETs formation via two key virulence factors: the type III secretion system (*bsaZ* and *bsaQ*) and capsular polysaccharide encoded by the *wcb* operon. Mutant strains lacking these components elicit stronger NETs responses and are more efficiently cleared by neutrophils compared to the wild-type strain (47). Here, we found that neutrophils infected with the Δ*sodA* strain of SS2 produced significantly higher levels of NETs than those infected with the WT strain (Fig. 3A through D). In contrast, the complemented strain restored NETs formation to levels comparable with WT, demonstrating that *sodA* suppresses NETs induction through its regulation of ROS production. Consistently, the Δ*sodA* strain exhibited reduced survival in neutrophils, which was restored by DNase I treatment, indicating a NETs-dependent killing mechanism (Fig. 4A and B). Taken together, the accumulation of ROS may promote innate immune activation in host cells. These findings further support the role of *sodA* in promoting SS2 immune evasion by modulating ROS levels and suppressing neutrophil NETs-mediated bactericidal activity.

NETs formation is a complex, multi-step process involving chromatin decondensation, ROS signaling, and membrane disruption (48). Recent studies have highlighted the central role of mitochondria in this process, particularly through the generation of mtROS, the regulation of membrane potential, and the release of mitochondrial DNA (49, 50). In this study, we found that *sodA* deficiency in SS2-infected neutrophils led to elevated ROS levels and significant disruption of mitochondrial membrane potential, suggesting that mitochondrial dysfunction contributes to enhanced NETs formation. On the other hand, accumulating evidence indicates that GSDMD, a pore-forming effector of pyroptosis, also plays a role in NETs formation. GSDMD-N, the cleaved active fragment, has been shown to insert into mitochondrial membranes, triggering mitochondrial damage, increased ROS production, and the release of pro-inflammatory signals (51, 52). In our study, we further observed that *sodA* deletion significantly increased the expression of GSDMD-N in neutrophils and promoted its colocalization with mitochondria, indicating that the GSDMD-mitochondria axis may be involved in *sodA*-mediated regulation of NETs. Taken together, these findings suggest that *sodA* contributes to immune evasion by scavenging ROS and maintaining mitochondrial integrity, thereby suppressing GSDMD activation and limiting NETs formation during SS2 infection.

It is worth noting that the attenuation observed in the *sodA* mutant may not solely result from impaired ROS detoxification in host cells. Previous studies have suggested that *sodA* deficiency disrupts intracellular redox homeostasis in *S. suis*, which could secondarily affect the expression of other virulence-associated genes (25). Such a redox imbalance may alter bacterial metabolism, membrane integrity, or signaling pathways, thereby contributing to the reduced virulence observed *in vivo*. Although our present study focused on the neutrophil response, these findings collectively suggest that *sodA* plays a multifaceted role in maintaining oxidative balance and virulence regulation in *S. suis*.

In summary, our study reveals that *sodA* plays a critical role in SS2 evasion of neutrophil-mediated immunity by limiting ROS accumulation, preserving mitochondrial membrane integrity, and suppressing GSDMD-N-driven NETs formation. These findings provide new mechanistic insight into the interface between bacterial oxidative stress defense and host innate immunity. While the precise regulatory interactions between *sodA*, mitochondrial dynamics, and GSDMD activation remain to be fully elucidated, our results highlight *sodA* as a potential target for enhancing host defense against SS2 infection.

## SUPPLEMENTAL MATERIAL

Figures S1 and S2 spectrum.01901-25-s0001.docx Figure S1: Validation of sodA transcription in WT, ΔsodA, and complemented (CΔsodA) strains. Figure S2: Viability and purity assessment of isolated mouse neutrophils.

Table S1 spectrum.01901-25-s0002.docx Primers used in this study.

ASM does not own the copyrights to Supplemental Material that may be linked to, or accessed through, an article. The authors have granted ASM a non-exclusive, world-wide license to publish the Supplemental Material files. Please contact the corresponding author directly for reuse.

## References

[1] Kerdsin A. 2022. Human Streptococcus suis infections in Thailand: epidemiology, clinical features, genotypes, and susceptibility. TropicalMed 7:359. doi:10.3390/tropicalmed7110359 PMC9695567 36355901

[2] Argirova P, Kalchev Y, Baltadzhiev I, Stoycheva M, Murdjeva M. 2023. Streptococcus zooepidemicus meningitis in an HIV-positive horse breeder patient: a case study and literature review. Infect Dis Rep 15:527–534. doi:10.3390/idr15050052 37736999 PMC10514876

[3] Payen S, Roy D, Okura M, Segura M, Gottschalk M. 2023. Study of the role of lipoprotein maturation enzymes in the pathogenesis of the infection caused by the Streptococcus suis serotype 2 sequence type 25 north American prototype strain. Pathogens 12:1325. doi:10.3390/pathogens12111325 38003790 PMC10675726

[4] Feng Y, Zhang H, Ma Y, Gao GF. 2010. Uncovering newly emerging variants of Streptococcus suis, an important zoonotic agent. Trends Microbiol 18:124–131. doi:10.1016/j.tim.2009.12.003 20071175

[5] Xi H, Fu Y, Chen C, Feng X, Han W, Gu J, Ji Y. 2023. Aerococcus viridans phage lysin AVPL had lytic activity against Streptococcus suis in a mouse bacteremia model. Int J Mol Sci 24:3390. doi:10.3390/ijms242316670 38068990 PMC10706753

[6] Guo G, Zhang Y, Wei D, Wang Z, Li Q, Yu Y, Zhang W. 2024. Contribution of nadR to the cell growth and virulence of Streptococcus suis serotype 2. Vet Microbiol 288:109928. doi:10.1016/j.vetmic.2023.109928 38056180

[7] Ding D, Li N, Ge Y, Wu H, Yu J, Qiu W, Fang F. 2024. Current status of superoxide dismutase 2 on oral disease progression by supervision of ROS. Biomed Pharmacother 175:116605. doi:10.1016/j.biopha.2024.116605 38688168

[8] Zhao H, Zhang R, Yan X, Fan K. 2021. Superoxide dismutase nanozymes: an emerging star for anti-oxidation. J Mater Chem B 9:6939–6957. doi:10.1039/d1tb00720c 34161407

[9] Krehenbrink M, Edwards A, Downie JA. 2011. The superoxide dismutase SodA is targeted to the periplasm in a SecA-dependent manner by a novel mechanism. Mol Microbiol 82:164–179. doi:10.1111/j.1365-2958.2011.07803.x 21854464

[10] Abuaita BH, Schultz TL, O’Riordan MX. 2018. Mitochondria-derived vesicles deliver antimicrobial reactive oxygen species to control phagosome-localized Staphylococcus aureus. Cell Host Microbe 24:625–636. doi:10.1016/j.chom.2018.10.005 30449314 PMC7323595

[11] Sun XY, Deng J, Zhang C, Fung SY, Siu KL, Cheng YY, Ye L, Qin J, Wang K, Qu JX, Gao W, Wang F, Jin DY, Yang L. 2024. Superoxide dismutase A (SodA) is a c-di-GMP effector protein governing oxidative stress tolerance in Stenotrophomonas maltophilia. Microbiol Res 278:127535. doi:10.1016/j.micres.2023.127535 37922698

[12] Okeke EB, Louttit C, Fry C, Najafabadi AH, Han K, Nemzek J, Moon JJ. 2020. Inhibition of neutrophil elastase prevents neutrophil extracellular trap formation and rescues mice from endotoxic shock. Biomaterials 238:119836. doi:10.1016/j.biomaterials.2020.119836 32045782 PMC7075277

[13] Bleuzé M, Gottschalk M, Segura M. 2021. Neutrophils in Streptococcus suis infection: from host defense to pathology. Microorganisms 9:2392. doi:10.3390/microorganisms9112392 34835517 PMC8624082

[14] Delgado-Rizo V, Martínez-Guzmán MA, Iñiguez-Gutierrez L, García-Orozco A, Alvarado-Navarro A, Fafutis-Morris M. 2017. Neutrophil extracellular traps and its implications in inflammation: an overview. Front Immunol 8:81. doi:10.3389/fimmu.2017.00081 28220120 PMC5292617

[15] Sun Q, Li N, Jia L, Guo W, Jiang H, Liu B, Bao C, Liu M, Huang J, Lei L. 2020. Ribosomal protein SA-positive neutrophil elicits stronger phagocytosis and neutrophil extracellular trap formation and subdues pro-inflammatory cytokine secretion against Streptococcus suis serotype 2 infection. Front Immunol 11:585399. doi:10.3389/fimmu.2020.585399 33603733 PMC7884477

[16] Xia X, Qin W, Zhu H, Wang X, Jiang J, Hu J. 2019. How Streptococcus suis serotype 2 attempts to avoid attack by host immune defenses. J Microbiol Immunol Infect 52:516–525. doi:10.1016/j.jmii.2019.03.003 30954397

[17] Azzouz D, Palaniyar N. 2024. How do ROS induce NETosis? Oxidative DNA damage, DNA repair, and chromatin decondensation. Biomolecules 14:1307. doi:10.3390/biom14101307 39456240 PMC11505619

[18] Chen KW, Monteleone M, Boucher D, Sollberger G, Ramnath D, Condon ND, von Pein JB, Broz P, Sweet MJ, Schroder K. 2018. Noncanonical inflammasome signaling elicits gasdermin D–dependent neutrophil extracellular traps. Sci Immunol 3:eaar6676. doi:10.1126/sciimmunol.aar6676 30143554

[19] Miao R, Jiang C, Chang WY, Zhang H, An J, Ho F, Chen P, Zhang H, Junqueira C, Amgalan D, et al.. 2023. Gasdermin D permeabilization of mitochondrial inner and outer membranes accelerates and enhances pyroptosis. Immunity 56:2523–2541. doi:10.1016/j.immuni.2023.10.004 37924812 PMC10872579

[20] Boilard E, Fortin PR. 2016. Connective tissue diseases: mitochondria drive NETosis and inflammation in SLE. Nat Rev Rheumatol 12:195–196. doi:10.1038/nrrheum.2016.24 26935279

[21] Lood C, Blanco LP, Purmalek MM, Carmona-Rivera C, De Ravin SS, Smith CK, Malech HL, Ledbetter JA, Elkon KB, Kaplan MJ. 2016. Neutrophil extracellular traps enriched in oxidized mitochondrial DNA are interferogenic and contribute to lupus-like disease. Nat Med 22:146–153. doi:10.1038/nm.4027 26779811 PMC4742415

[22] Chen KW, Demarco B, Broz P. 2020. Beyond inflammasomes: emerging function of gasdermins during apoptosis and NETosis. EMBO J 39:e103397. doi:10.15252/embj.2019103397 31793683 PMC6960442

[23] Ma F, Chang X, Wang G, Zhou H, Ma Z, Lin H, Fan H. 2018. Streptococcus suis serotype 2 stimulates neutrophil extracellular traps formation via activation of p38 MAPK and ERK1/2. Front Immunol 9:2854. doi:10.3389/fimmu.2018.02854 30581435 PMC6292872

[24] Fang L, Shen H, Tang Y, Fang W. 2015. Superoxide dismutase of Streptococcus suis serotype 2 plays a role in anti-autophagic response by scavenging reactive oxygen species in infected macrophages. Vet Microbiol 176:328–336. doi:10.1016/j.vetmic.2015.02.006 25726301

[25] Tang Y, Zhang X, Wu W, Lu Z, Fang W. 2012. Inactivation of the sodA gene of Streptococcus suis type 2 encoding superoxide dismutase leads to reduced virulence to mice. Vet Microbiol 158:360–366. doi:10.1016/j.vetmic.2012.02.028 22424868

[26] Parker HA, Magon NJ, Green JN, Hampton MB, Winterbourn CC. 2014. Analysis of neutrophil bactericidal activity. Methods Mol Biol 1124:291–306. doi:10.1007/978-1-62703-845-4_19 24504960

[27] Ma F, Yi L, Yu N, Wang G, Ma Z, Lin H, Fan H. 2017. Streptococcus suis serotype 2 biofilms inhibit the formation of neutrophil extracellular traps. Front Cell Infect Microbiol 7:86. doi:10.3389/fcimb.2017.00086 28373968 PMC5357632

[28] Dömer D, Walther T, Möller S, Behnen M, Laskay T. 2021. Neutrophil extracellular traps activate proinflammatory functions of human neutrophils. Front Immunol 12:636954. doi:10.3389/fimmu.2021.636954 34168641 PMC8217666

[29] Brinkmann V, Goosmann C, Kühn LI, Zychlinsky A. 2012. Automatic quantification of in vitro NET formation. Front Immunol 3:413. doi:10.3389/fimmu.2012.00413 23316198 PMC3540390

[30] Metzler KD, Goosmann C, Lubojemska A, Zychlinsky A, Papayannopoulos V. 2014. A myeloperoxidase-containing complex regulates neutrophil elastase release and actin dynamics during NETosis. Cell Rep 8:883–896. doi:10.1016/j.celrep.2014.06.044 25066128 PMC4471680

[31] Xu G, Guo Z, Liu Y, Yang Y, Lin Y, Li C, Huang Y, Fu Q. 2022. Gasdermin D protects against Streptococcus equi subsp. zooepidemicus infection through macrophage pyroptosis. Front Immunol 13:100592510. doi:10.3389/fimmu.2022.1005925 PMC9614658 36311722

[32] Herb M, Schramm M. 2021. Functions of ROS in macrophages and antimicrobial immunity. Antioxidants (Basel) 10:313. doi:10.3390/antiox10020313 33669824 PMC7923022

[33] Willems P, Rossignol R, Dieteren CEJ, Murphy MP, Koopman WJH. 2015. Redox homeostasis and mitochondrial dynamics. Cell Metab 22:207–218. doi:10.1016/j.cmet.2015.06.006 26166745

[34] Feng Y, Zhang H, Wu Z, Wang S, Cao M, Hu D, Wang C. 2014. Streptococcus suis infection: an emerging/reemerging challenge of bacterial infectious diseases? Virulence 5:477–497. doi:10.4161/viru.28595 24667807 PMC4063810

[35] Segura M, Fittipaldi N, Calzas C, Gottschalk M. 2017. Critical Streptococcus suis virulence factors: are they all really critical? Trends Microbiol 25:585–599. doi:10.1016/j.tim.2017.02.005 28274524

[36] Baz AA, Hao H, Lan S, Li Z, Liu S, Chen S, Chu Y. 2024. Neutrophil extracellular traps in bacterial infections and evasion strategies. Front Immunol 15:1357967. doi:10.3389/fimmu.2024.1357967 38433838 PMC10906519

[37] Dan Dunn J, Alvarez LA, Zhang X, Soldati T. 2015. Reactive oxygen species and mitochondria: a nexus of cellular homeostasis. Redox Biol 6:472–485. doi:10.1016/j.redox.2015.09.005 26432659 PMC4596921

[38] Xie F, Zan Y, Zhang Y, Zheng N, Yan Q, Zhang W, Zhang H, Jin M, Chen F, Zhang X, Liu S. 2019. The cysteine protease ApdS from Streptococcus suis promotes evasion of innate immune defenses by cleaving the antimicrobial peptide cathelicidin LL-37. J Biol Chem 294:17962–17977. doi:10.1074/jbc.RA119.009441 31619521 PMC6879338

[39] Brockmeier SL, Loving CL, Eberle KC, Hau SJ, Mou KT, Kehrli ME Jr. 2019. Administration of granulocyte-colony stimulating factor (G-CSF) to pigs results in a longer mean survival time after exposure to Streptococcus suis. Vet Microbiol 231:116–119. doi:10.1016/j.vetmic.2019.03.010 30955798

[40] Hu X, Lu Y, Yu X, Jia K, Xiong Q, Fang R. 2024. The suppressive role of NLRP6 in host defense against Streptococcus suis infection. Vet Microbiol 296:110166. doi:10.1016/j.vetmic.2024.110166 38968694

[41] Meng Y, Lin S, Niu K, Ma Z, Lin H, Fan H. 2023. Vimentin affects inflammation and neutrophil recruitment in airway epithelium during Streptococcus suis serotype 2 infection. Vet Res 54:7. doi:10.1186/s13567-023-01135-3 36717839 PMC9885403

[42] Liu M, Zhu H, Li J, Garcia CC, Feng W, Kirpotina LN, Hilmer J, Tavares LP, Layton AW, Quinn MT, Bothner B, Teixeira MM, Lei B. 2012. Group A Streptococcus secreted esterase hydrolyzes platelet-activating factor to impede neutrophil recruitment and facilitate innate immune evasion. PLoS Pathog 8:e1002624. doi:10.1371/journal.ppat.1002624 22496650 PMC3320582

[43] Zhong G, Guo Y, Gong X, Xu M, Wang Q, Wu M, Zhang X, Liang Y, Zhao W, Wang H, Ye J. 2023. Enhanced glycolysis by ATPIF1 gene inactivation increased the anti-bacterial activities of neutrophils through induction of ROS and lactic acid. Biochim Biophys Acta Mol Basis Dis 1869:166820. doi:10.1016/j.bbadis.2023.166820 37558010

[44] Zhang XW, An MX, Huang ZK, Ma L, Zhao D, Yang Z, Shi JX, Liu DX, Li Q, Wu AH, Chen YH, Zhao WD. 2023. Lpp of Escherichia coli K1 inhibits host ROS production to counteract neutrophil-mediated elimination. Redox Biol 59:102588. doi:10.1016/j.redox.2022.102588 36592568 PMC9823224

[45] Niu A, Bian WP, Feng SL, Pu SY, Wei XY, Yang YF, Song LY, Pei DS. 2021. Role of manganese superoxide dismutase (Mn-SOD) against Cr(III)-induced toxicity in bacteria. J Hazard Mater 403:123604. doi:10.1016/j.jhazmat.2020.123604 32781281

[46] Badilla-Vargas L, Pereira R, Molina-Mora JA, Alape-Girón A, Flores-Díaz M. 2023. Clostridium perfringens phospholipase C, an archetypal bacterial virulence factor, induces the formation of extracellular traps by human neutrophils. Front Cell Infect Microbiol 13:1278718. doi:10.3389/fcimb.2023.1278718 37965263 PMC10641792

[47] Riyapa D, Buddhisa S, Korbsrisate S, Cuccui J, Wren BW, Stevens MP, Ato M, Lertmemongkolchai G. 2012. Neutrophil extracellular traps exhibit antibacterial activity against Burkholderia pseudomallei and are influenced by bacterial and host factors. Infect Immun 80:3921–3929. doi:10.1128/IAI.00806-12 22927051 PMC3486034

[48] Storisteanu DML, Pocock JM, Cowburn AS, Juss JK, Nadesalingam A, Nizet V, Chilvers ER. 2017. Evasion of neutrophil extracellular traps by respiratory pathogens. Am J Respir Cell Mol Biol 56:423–431. doi:10.1165/rcmb.2016-0193PS 27854516 PMC5449512

[49] Rungelrath V, Kobayashi SD, DeLeo FR. 2020. Neutrophils in innate immunity and systems biology-level approaches. Wiley Interdiscip Rev Syst Biol Med 12:e1458. doi:10.1002/wsbm.1458 31218817 PMC6898734

[50] Vorobjeva NV, Chernyak BV. 2020. NETosis: molecular mechanisms, role in physiology and pathology. Biochemistry (Mosc) 85:1178–1190. doi:10.1134/S0006297920100065 33202203 PMC7590568

[51] Kuang L, Wu Y, Shu J, Yang J, Zhou H, Huang X. 2024. Pyroptotic macrophage-derived microvesicles accelerate formation of neutrophil extracellular traps via GSDMD-N-expressing mitochondrial transfer during sepsis. Int J Biol Sci 20:733–750. doi:10.7150/ijbs.87646 38169726 PMC10758106

[52] Miao N, Kang Z, Wang Z, Yu W, Liu T, Kong LZ, Zheng Y, Ding C, Zhang Z, Zhong C, Fang Q, Li K. 2025. Mitochondrial reactive oxygen species promote cancer metastasis and tumor microenvironment immunosuppression through gasdermin D. Cell Death Discov 11:219. doi:10.1038/s41420-025-02516-7 40324993 PMC12053750

