# Supplementary material for: SodA promotes immune evasion of Streptococcus suis by suppressing ROS accumulation and GSDMD-mediated mitochondrial disruption in neutrophils
Source: Microbiol Spectr. 2025 Nov 26;14(1):e01901-25. doi: 10.1128/spectrum.01901-25 (PMC12772235; doi:10.1128/spectrum.01901-25)
Supplement: Figures S1 and S2 — Figure S1: Validation of sodA transcription in WT, ΔsodA, and complemented (CΔsodA) strains. Figure S2: Viability and purity assessment of isolated mouse neutrophils. [file spectrum.01901-25-s0001.docx]

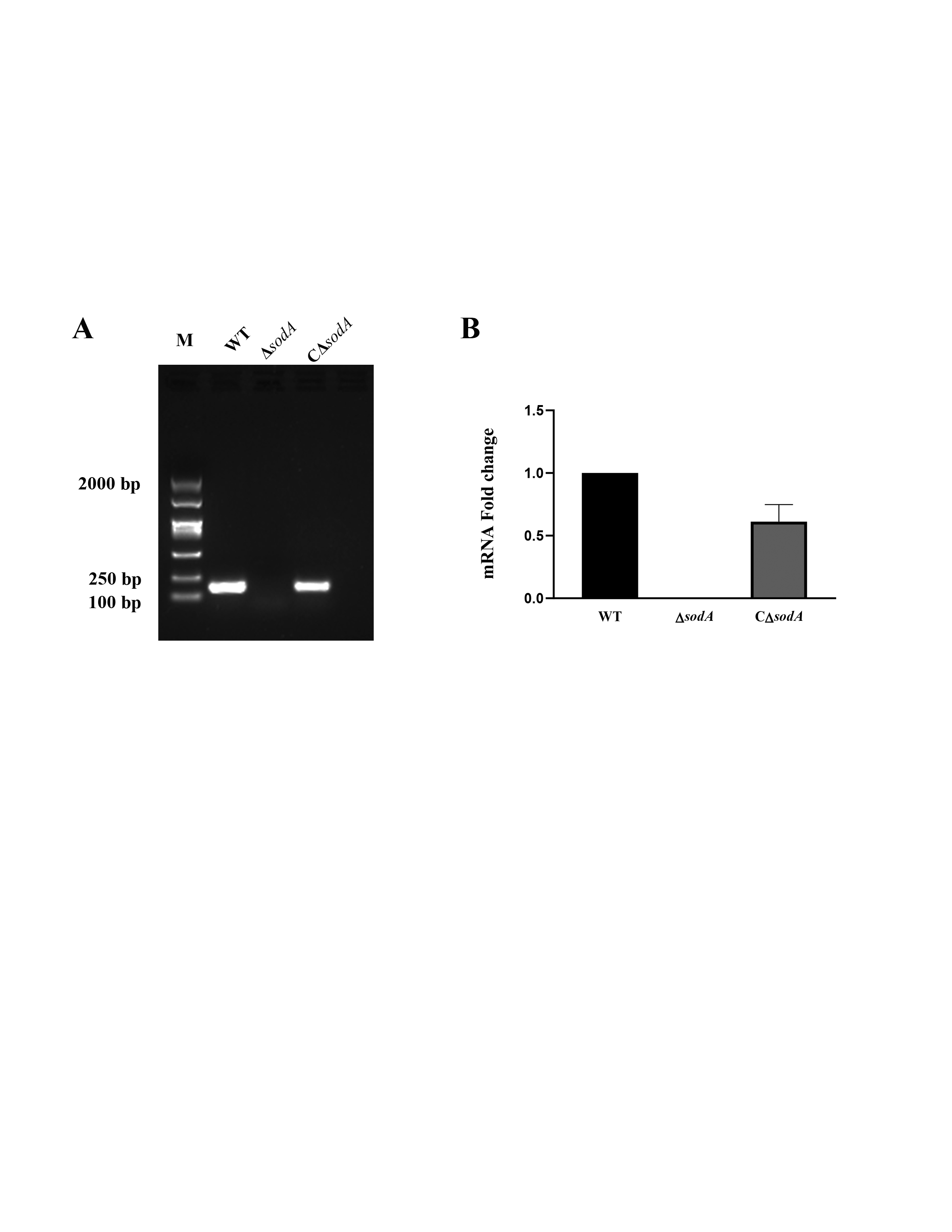


**Fig. S1. Validation of *sodA* transcription in WT, Δ*sodA*, and complemented (CΔ*sodA*) strains.**

Total RNA was extracted from logarithmic-phase cultures of WT, Δ*sodA*, and CΔ*sodA* strains using TRIzol reagent, and cDNA was synthesized by reverse transcription. (A) RT-PCR analysis of *sodA* transcription. Agarose gel electrophoresis showed that *sodA* was transcribed normally in the complemented strain (CΔ*sodA*), while no transcription was detected in the Δ*sodA* mutant. (B) Quantitative real-time PCR (qPCR) analysis of *sodA* expression. The transcription level of *sodA* was normalized to the internal control gene 16S rRNA, with WT expression used as the reference for relative quantification.


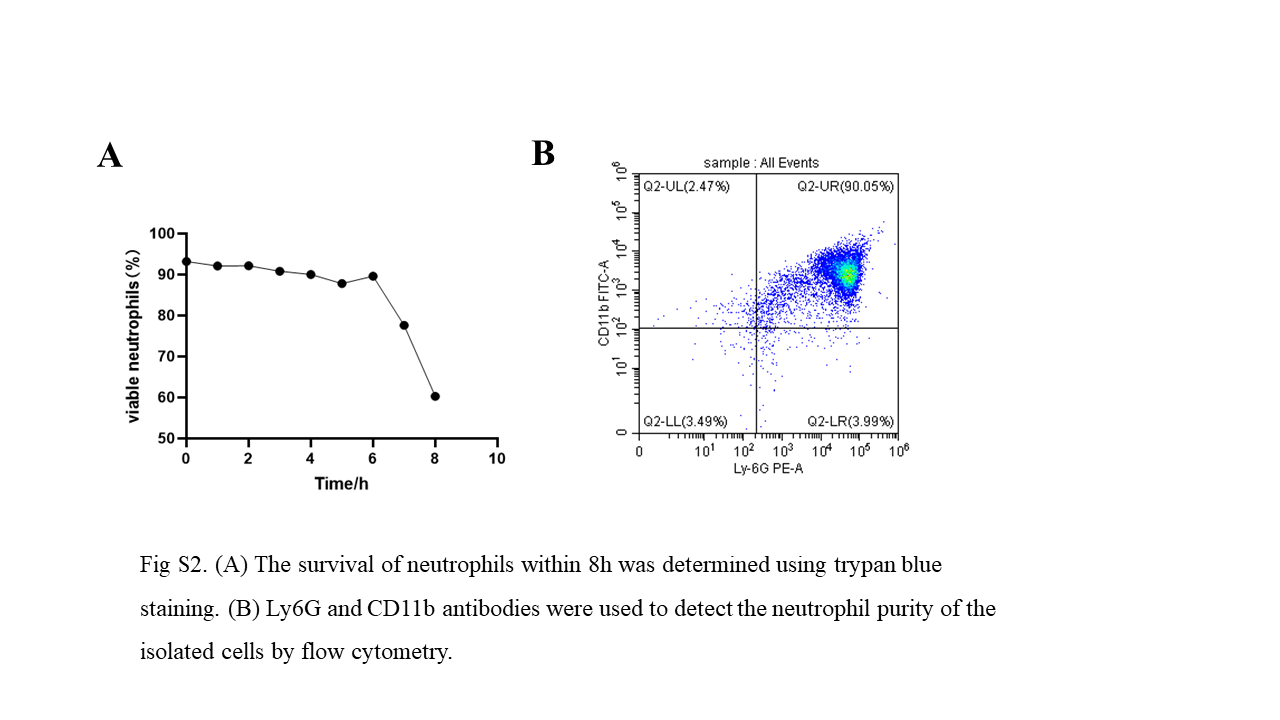


**Fig. S2. Viability and purity assessment of isolated mouse neutrophils.** (A) Neutrophil viability was assessed within 8 hours after isolation using trypan blue exclusion staining. (B) Neutrophil purity was determined by flow cytometry using Ly6G and CD11b surface markers. The majority of the isolated cells were Ly6G⁺/CD11b⁺, indicating a purity greater than 90%.
