# Supplementary material for: SodA promotes immune evasion of Streptococcus suis by suppressing ROS accumulation and GSDMD-mediated mitochondrial disruption in neutrophils
Source: Microbiol Spectr. 2025 Nov 26;14(1):e01901-25. doi: 10.1128/spectrum.01901-25 (PMC12772235; doi:10.1128/spectrum.01901-25)
Supplement: Table S1 — Primers used in this study. [file spectrum.01901-25-s0002.docx]

| Primer name | Sequence (5′–3′) | Restriction site | Expected size (bp) | Descrition |
| --- | --- | --- | --- | --- |
| CsodA-F | GGACAGC**GAATTC**ATCTGCTAAAACAGCTT | ***EcoRI*** | 986 | Construction of sodA complemented strain |
| CsodA-R | AGTATA**GGATCC**AACGAAAATGACAATTATTT | ***BamHI*** |  |  |
| qSodA-F | TAGTTTGGACGGACATTGCGGTAG | ***/*** | 167 | qPCR validation of sodA transcription |
| qSodA-R | TTCGGTTCAGGTTGGGCTTTCTT | ***/*** |  |  |
| 16SrRNA-F | TAGGGTTTCTCTTCGGAGCATCG | ***/*** | 123 | qPCR internal control (16S rRNA) |
| 16SrRNA-R | AACTGAATGATGGCAACT | ***/*** |  |  |

**Table S1. Primers used in this study**
